# Functional characterization of a monoclonal antibody epitope using a lambda phage display-deep sequencing platform

**DOI:** 10.1038/srep31458

**Published:** 2016-08-17

**Authors:** Maria Domina, Veronica Lanza Cariccio, Salvatore Benfatto, Mario Venza, Isabella Venza, Erica Borgogni, Flora Castellino, Angelina Midiri, Roberta Galbo, Letizia Romeo, Carmelo Biondo, Vega Masignani, Giuseppe Teti, Franco Felici, Concetta Beninati

**Affiliations:** 1 Scylla Biotech Srl, Messina, Italy; 2 Department of Human Pathology, University of Messina, Messina, Italy; 3 Department of Clinical and Experimental Medicine, University of Messina, Messina, Italy; 4 GSK Vaccines, Siena, Italy; 5 Department of Biological, Chemical and Environmental Sciences, University of Messina, Messina, Italy; 6 Charybdis Vaccines Srl, Messina, Italy; 7 Department of Biosciences and Territory, University of Molise, Pesche, Isernia, Italy

## Abstract

We have recently described a method, named PROFILER, for the identification of antigenic regions preferentially targeted by polyclonal antibody responses after vaccination. To test the ability of the technique to provide insights into the functional properties of monoclonal antibody (mAb) epitopes, we used here a well-characterized epitope of meningococcal factor H binding protein (fHbp), which is recognized by mAb 12C1. An fHbp library, engineered on a lambda phage vector enabling surface expression of polypeptides of widely different length, was subjected to massive parallel sequencing of the phage inserts after affinity selection with the 12C1 mAb. We detected dozens of unique antibody-selected sequences, the most enriched of which (designated as FrC) could largely recapitulate the ability of fHbp to bind mAb 12C1. Computational analysis of the cumulative enrichment of single amino acids in the antibody-selected fragments identified two overrepresented stretches of residues (H248-K254 and S140-G154), whose presence was subsequently found to be required for binding of FrC to mAb 12C1. Collectively, these results suggest that the PROFILER technology can rapidly and reliably identify, in the context of complex conformational epitopes, discrete “hot spots” with a crucial role in antigen-antibody interactions, thereby providing useful clues for the functional characterization of the epitope.

Epitope mapping is a fundamental step in the study of macromolecular interactions, particularly in the development of vaccines, drugs and diagnostics^1^. For example, this approach can provide in-depth analysis of the interaction site between a drug and its target or, in the case of vaccines, of the mechanisms underlying anti-pathogen immunity. NMR spectroscopy and X-ray co-crystallography are gold standards in epitope mapping, but are very laborious, costly and not always applicable. Chemical crosslinking followed by mass spectrometry analysis has developed into a reliable tool for characterizing antigen epitopes and, in general, structural details of functional complexes in solution^2^,^3^. However, this technique also involves considerable time and expertise. Array-based scanning of overlapping synthetic oligopeptides is a simpler and more widely used method, which is useful in the characterization of linear epitopes. However, this technique has limited ability to detect conformational epitopes, which represent up to 90% of all antigenic determinants of a protein^4^,^5^,^6^. Therefore, there is presently a need for rapid and accurate epitope mapping techniques that can keep pace with currently available methods for the isolation of increasingly larger numbers of potentially useful mAbs. The phage display technology, in which artificial oligopeptides or natural protein fragments are expressed on the phage surface in fusion with coat proteins, can also be used for epitope mapping, by virtue of its efficiency in selecting antibody ligands, low costs and rapidity^7^,^8^,^9^.

The most common approach to this technique involves the use of filamentous phage M13 vectors expressing random oligopeptides as fusions to coat proteins. Screening of such libraries may allow affinity selection of peptides matching short stretches of linear continuous epitopes. However, unambiguous identification of epitopes that are longer or adopt structural conformation often requires the use of gene fragment libraries engineered on phage vectors that can tolerate expression of large protein domains^10^,^11^. We have successfully employed one of such vectors, based on a lambda phage, for antigen discovery using genomic libraries obtained from bacterial pathogens^12^,^13^. However, the ability of this system to express a wide variety of protein domains spanning several hundred residues, as well as oligopeptides^10^,^14^, renders it ideally suited also for epitope mapping. We have recently combined the efficiency of this antigen display system with the power of next generation sequencing into a platform allowing the characterization of antibody repertoires in polyclonal antibody mixtures such as serum samples from vaccinated individuals. The technology, named PROFILER, (standing for “Phage-based Representation OF ImmunoLigand Epitope Repertoire”), can provide a detailed immunodominance profile of the antigen regions targeted by an antibody response in a two-day frame^15^. To explore the potential use of this platform in mapping monoclonal antibodies (mAb) epitopes, in the present study we chose to use, as a model system, a mAb, designated as 12C1, whose binding site has been fully characterized from the structural viewpoint. This mAb binds to a complex epitope on a variant of factor H binding protein (fHbp var1), an important component of human vaccines directed against group B *Neisseria meningitidis*^16^. It was found here that the PROFILER technology could reliably identify the epitope-containing antigen region and provide, in addition, clues for the functional characterization of the epitope, including the identification of the minimal sequence of fHbp that could still strongly bind mAb 12C1.

## Results and Discussion

### Construction and characterization of an antigen-specific lambda phage-displayed library

**Figure 1:**
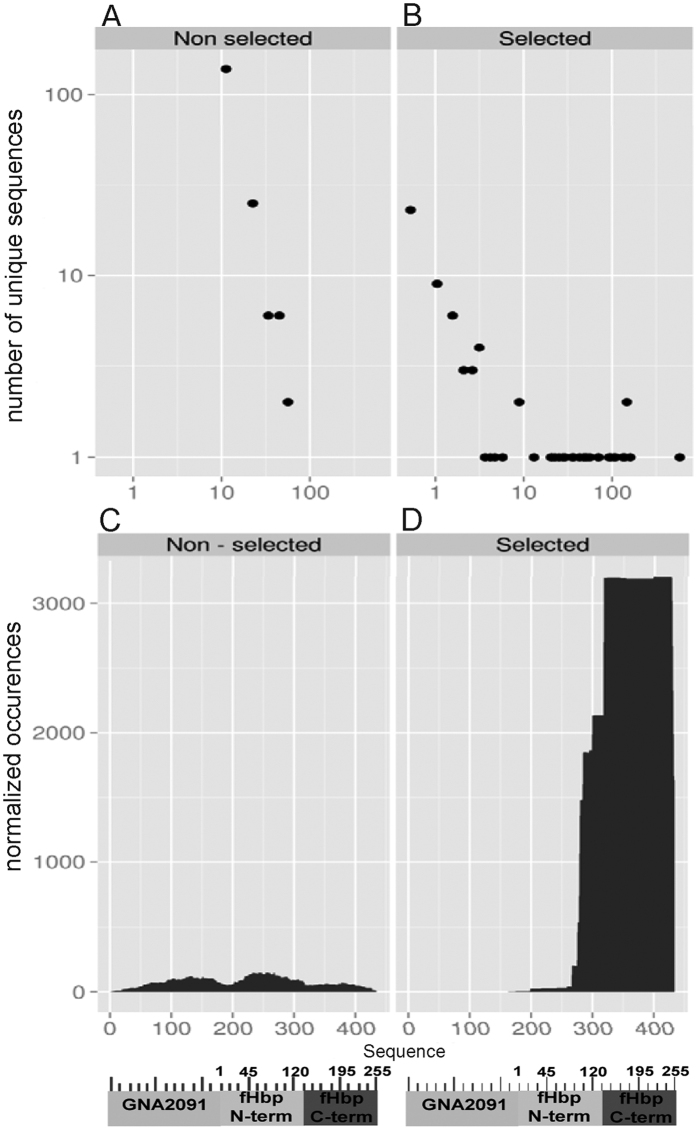
Upper panels: diversity of the GNA2091-fHbp lambda phage-displayed library before and after affinity selection with the 12C1 mAb. Shown is the abundance of unique “natural frame” sequences in the unselected (panel A) and affinity-selected (panel B) libraries. Each point represents the number of unique sequences (vertical axis) displaying the number of copies indicated in the horizontal axis. Lower panels: Enrichment of “natural frame” GNA2091-fHbp phage inserts after affinity selection with the 12C1 mAb. Each graph reports the cumulative occurrence, per single aminoacid position, of predicted “natural frame” sequences before (panel C) and after (panel D) affinity selection with the 12C1 mAb. The horizontal axis reports the aa sequence corresponding to the GNA2091-fHbp fusion gene used to engineer the library. The occurrence of each “natural frame” sequence was normalized over the total number of sequences. A schematic representation of the GNA2091-fHbp fusion protein is reported below the horizontal axis.

We employed, for the construction of a fHbp var1-specific library, a previously described lambda phage vector^12^,^15^,^17^ using fragments of the gene encoding for the GNA2091-fHbp var-1 fusion protein included in the Bexsero^®^ vaccine. The quality and diversity of the library was characterized using a deep sequencing strategy that allowed the identification of both “empty” (i.e. wild type) and recombinant vector particles. Five percent of all sequences containing *GNA2091-fHbp* inserts were predicted to be “natural frame” i.e. to be expressed on the phage surface as fusions with capsid protein D. This percentage is close to the expected maximal 1/18 (5.6%) value, calculated as the probability that a gene fragment is randomly cloned as an insert in the natural frame at the N-terminus of the lambda phage capsid protein D encoding sequence^15^,^17^ (Supplementary Note S1). The fragments were diverse (Supplementary Fig. 1A) and evenly distributed along the sequence of the protein, with no major over- or under-representations of specific regions (Fig. 1C). Moreover, the length distribution of expressed authentic GNA2091-fHbp fragments had a mean value of 55 (49, median; 18, standard deviation) amino acids (aa). Therefore, the GNA2091-fHbp library characteristics were in agreement with its design.

### Identification of affinity-selected fHbp fragments by deep sequencing

**Figure 2:**
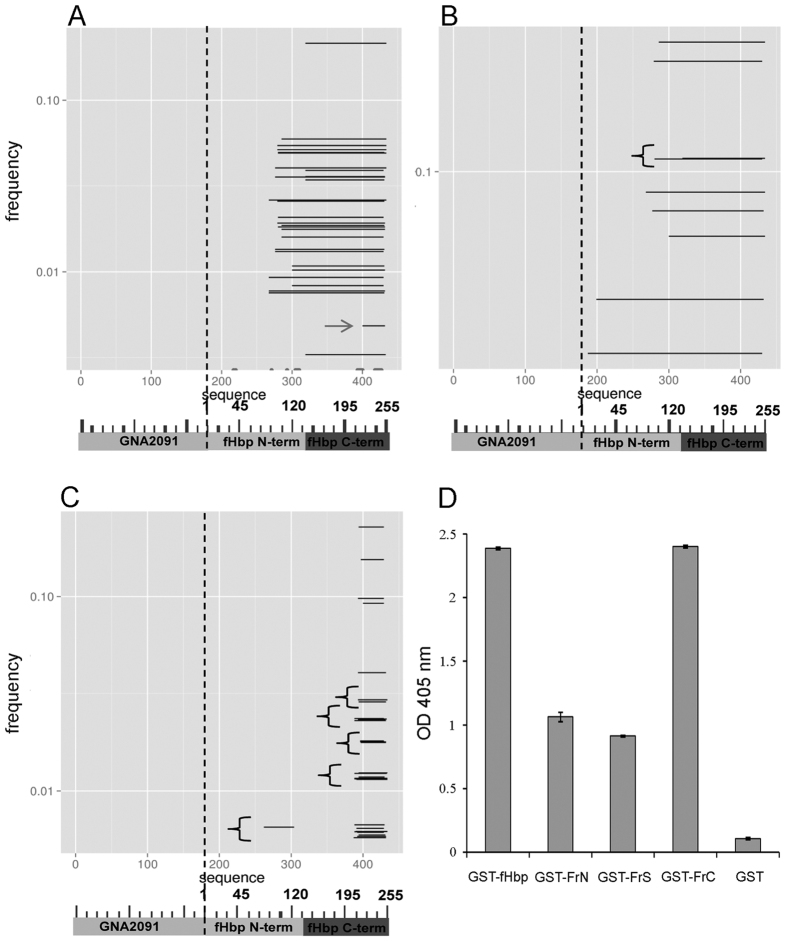
Enrichment of fHbp fragments after affinity-selection of phage-displayed libraries and immunoreactivity of selected antigenic fragments with the 12C1 mAb. Panel A shows the 30 most enriched fragments in the GNA2091-fHbp lambda library after selection, as identified by next generation sequencing. The small spots along the horizontal axis indicate the 23 contact points between mAb 12C1 and fHbp var 1, as previously determined by X-ray spectroscopy^16^ and the arrow identifies the shortest fragment. Panel B shows the most enriched fragments identified by Sanger sequencing of immunoreactive clones after immunoblotting in the same lambda library. Panel C shows the fragments identified by NGS sequencing in the filamentous phage library after affinity selection with mAb 12C1. Fragments with the same frequency were artificially separated for clarity of representation and are indicated by the curly brackets. A schematic representation of the GNA2091-fHbp fusion protein is reported below the horizontal axis. Panel D shows the results of an ELISA assay in which recombinant fHbp or its fragments, fused to GST, were immobilized on plastic wells followed by the addition of mAb 12C1 and enzyme-conjugated anti-mouse Ig. The ELISA assay was performed in triplicate and results are from one experiment representative of three producing similar data.

Phage clones obtained after two rounds of affinity selection with the 12C1 mAb were then subjected to deep sequencing using the MiSeq Illumina technology, as described above, yielding 5,172 “natural frame” reads. As it can be appreciated from Fig. 1A,B, library complexity decreased after selection to rapidly converge towards a relatively restricted number of unique sequences. Moreover, the frequency of “natural frame” fragments increased dramatically over the total number of sequences, as indicated by the black areas in Fig. 1C,D. Collectively, these data suggested that the phage populations expressing authentic antigenic fragments had been selectively enriched by mAb 12C1, while those carrying “not natural frame” or no antigenic inserts markedly decreased in numbers. As expected from the specificity of mAb 12C1 for fHbp, *GNA2091* sequences were not enriched by the affinity selection process (Fig. 1D). Moreover, the affinity-selected fragments were not evenly distributed along the entire length of fHbp, but rather clustered near the C-terminal portion of protein. Figure 2A shows the 30 most enriched fragments in the selected library, ranked by their frequency of occurrence. The most and least enriched of these fragments had frequency values of 0.21 and 0.03, respectively, among all “natural frame” sequences detected in the affinity-selected library. The most enriched fragment (S140-K254), encompassed almost the entire C-terminal beta-barrel domain (H138-Q255) of fHbp and contained 15 of the 23 contact points between fHbp var1 and mAb 12C1, as previously determined by X-ray spectroscopy^16^. Moreover, the S140-Q250 sequence was shared by all of the 30 most enriched fragments, with the exception of a relatively short aa fragment spanning residues S221-A253 (indicated by the arrow in Fig. 2A). Of note, this latter fragment resembled those recognized by the same mAb in our previous study using an M13 filamentous phage library in which antigenic fragments are displayed as fusions to coat protein VIII^16^. Therefore, it was of interest to ascertain whether the preferential identification of longer fragments in the present, relative to the previous, study was related to differences in the library vectors (i.e. lambda/D *vs*. M13/VIII phage display) or in the methods (i.e. sequencing of affinity-selected phage population *vs.* immunoscreening) used to detect antibody-interacting phage clones. To clarify this point, we subjected the affinity-selected lambda and M13 phage libraries to, respectively, immunoscreening and deep sequencing. Immunoscreening of the lambda library followed by Sanger sequencing of 82 positive clones identified only long (>115 aa-long) fragments (Fig. 2B). On the other hand, deep sequencing of the affinity-selected M13 library detected only short (<40 aa long) fragments (Fig. 2C). Thus, differences in the length of identified fragments were linked to intrinsic properties of the vectors used in library construction. Moreover, deep sequencing of the lambda library allowed the identification of a larger variety of affinity-selected fragments relative to the other method tested.

### Reactivity of the 12C1 mAb against recombinant fHbp fragments

**Figure 3:**
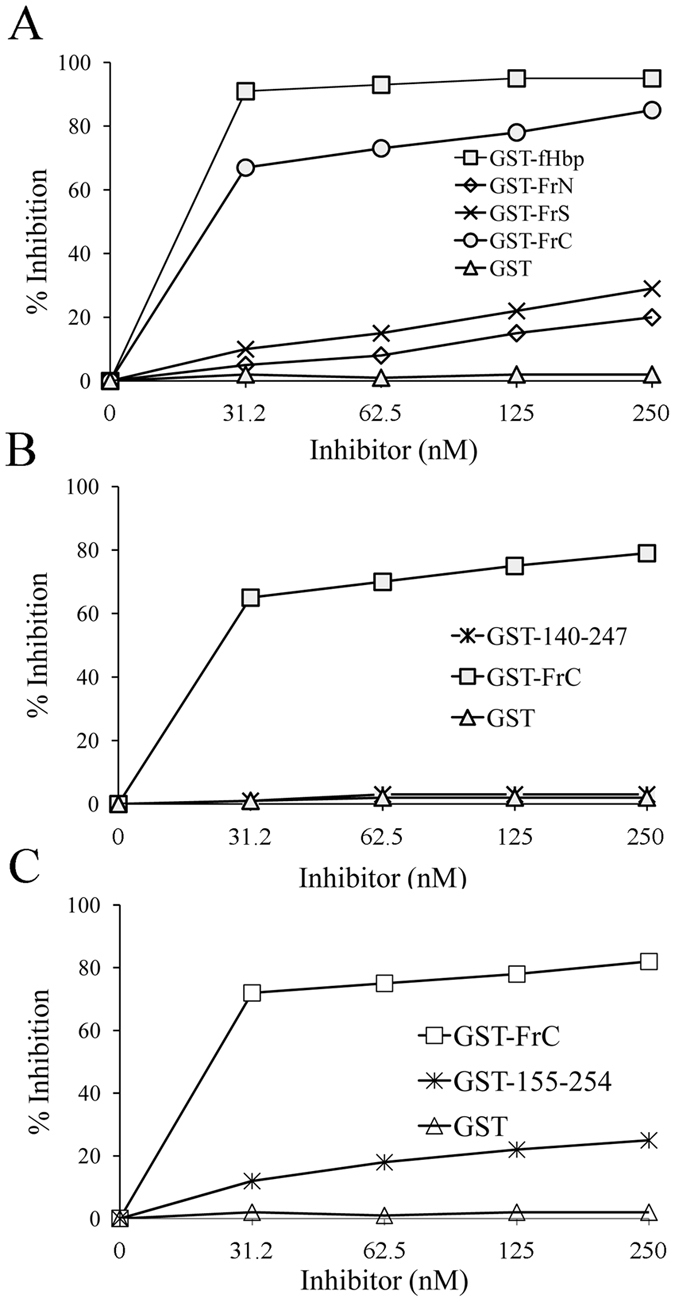
Competitive inhibition of binding of mAb 12C1 to fHbp by recombinant fHbp fragments. Plates were coated with the GST-fHbp recombinant protein and reacted with limiting amounts of 12C1 mAb in the presence of increasing concentrations of the indicated fragments (GST was used as a negative control). Data are from one experiment representative of three producing similar results.

In view of the above results, it was of interest to ascertain whether and to what extent the identified fragments were able to react with mAb 12C1 outside of the context of capsid phage proteins. To this end, we expressed two representative polypeptides, designated as GST-FrC (S140-K254) and GST-FrS (N215-K254) as fusions to gluthatione-S-transferase (GST). GST and GST-fHbp were used as negative and positive controls, respectively. We also tested a fragment, designated GST-FrN that mapped to an area (H26-A108) entirely comprised within the N-terminal domain of fHbp, although this or similar fragments were not significantly enriched by the affinity selection process (data not shown). When tested in an ELISA assay, in which the recombinant proteins were adsorbed to the solid phase, mAb 12C1 reacted strongly against GST-FrC and GST-fHbp, while only moderate binding was observed using GST-FrS or GST-FrN as coating antigens (Fig. 2D). These results were confirmed by a sensitive ELISA inhibition assay in which soluble fragments were used to inhibit binding of the 12C1 mAb to GST-fHbp, used as a coating antigen. Under these conditions, GST-FrC competed with immobilized GST-fHbp for mAb 12C1 binding almost as effectively as soluble GST-fHbp (Fig. 3A). In contrast, GST-FrS produced only slight inhibition of mAb 12C1 binding, even at high doses. Interestingly, GST-FrN also produced slight inhibition, which is compatible with the known presence of 3 contact points between fHbp and mAb 12C1 in this fragment^16^. These data suggested that the long fragments identified in the lambda display library, such as FrC, might better reconstitute the complex conformational epitope recognized by mAb12C1, relative to the considerably shorter fragments (e.g. FrS) identified in a previous study^16^.

### Functional requirements for binding to mAb 12C1

**Figure 4:**
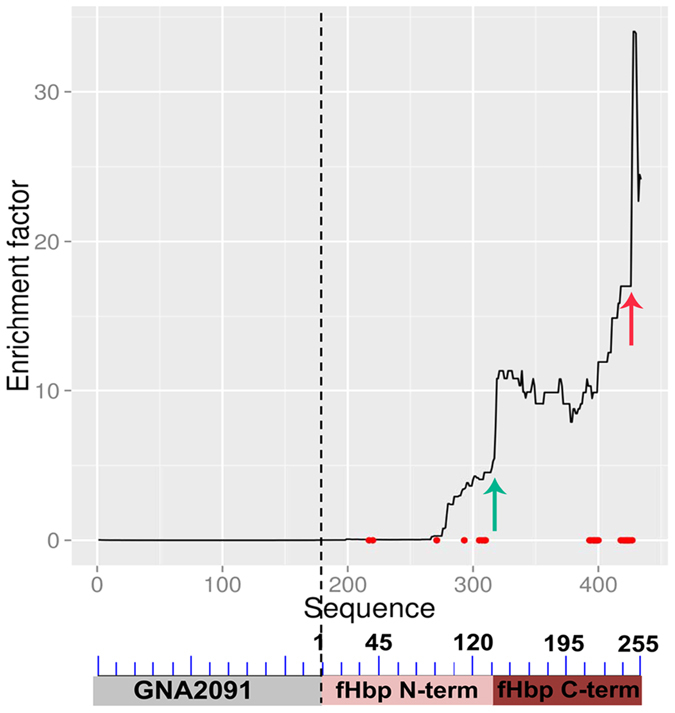
Cumulative “enrichment factor” values for each fHbp aa residue. Enrichment factor was calculated as the ratio between the occurrence of the residue in the affinity-selected phage population and its occurrence in the unselected library. The horizontal axis reports the sequence of the GNA2091-fHbp protein. Red spots on the horizontal axis indicate the 23 contact points between mAb 12C1 and fHbp var 1, as previously determined by X-ray spectroscopy^16^. The arrows indicate points of sudden increase in enrichment factor values. A schematic representation of the GNA2091-fHbp fusion protein is reported below the horizontal axis.

While our initial analysis was limited to 30 fragments for ease of comparison with traditional phage display approaches (Fig. 2), in further studies we sought to take advantage of the large variety of “natural frame” fragments identified by deep sequencing in the entire phage populations. We specifically asked whether overrepresentation of relatively short aa sequences after affinity selection might signal their requirement for binding to mAb 12C1. To this end, we plotted cumulative “enrichment factor” values (defined as the ratio between the occurrence of a given antigen fragment in the affinity-selected library and its occurrence in the unselected library; see ref. 15 and the Methods section) for each aa residue along the fHbp sequence. As shown in Fig. 4, the enrichment factor suddenly increased between R247 and H248 (as indicated by the red arrow) to peak at residues H248-L251. Based on this data, we sought to determine whether C-terminal truncation of FrC at R247 affected its ability to interact with mAb 12C1. Figure 3B shows that this was indeed the case, since a truncated form of GST-FrC (designated as GST-140-247) was no longer able to interact with mAb12C1 in the ELISA inhibition assay. Interestingly, the H248-K254 region, which was apparently required for binding of FrC to mAb 12C1, contains a contact point (H248) with mAb 12C1^16^. Thus, these data raised the possibility that abrupt changes in cumulative enrichment factor values along the antigen sequence might signal the presence of residues, or groups of residues, required for antibody binding. We further explored this possibility, having detected, between T139 and S140, an additional point of sudden increase in enrichment factor (indicated by the green arrow in Fig. 4). As in the previous example, we hypothesized that the region lying beyond this point, in the direction of increasing values, might play a role in antibody binding. To test this, we produced a C terminally truncated form of GST-FrC (designated as GST-155-254), lacking the region under scrutiny. As shown in Fig. 3C, the truncated fragment almost completely lost activity in the ELISA inhibition assay, indicating that the S140-G154 sequence is also required for optimal binding of FrC to mAb 12C1. Since contact points with mAb 12C1are not present in this region^16^, it is likely that the S140-G154 sequence promotes binding to mAb 12C1 by indirect mechanisms, likely by allowing the correct folding of FrC.

Next, we sought to more precisely assess the binding properties of these fragments by measuring their affinities for mAb 12C1 in comparison with the entire antigen. To this end, we used an ELISA assay^18^ that can provide reliable estimates of the equilibrium dissociation constant (K_D_) of antigen-antibody complexes in solution^19^. The binding affinities of mAb 12C1 for the fragments under study are reported in Table 1 and the methods used for K_D_ calculation are exemplified in Supplementary Figure 1. The full-length fHbp antigen displayed a high affinity for mAb 12C1, while the affinity of GST-FrC was moderately (23-fold) lower. In contrast, GST-FrS, GST-FrN or GST-155-254 (i.e. the N-terminally truncated form of FrC) displayed K_D_ values that were considerably (356- to 429-fold) lower than those of full-length fHbp (Table 1). No interaction was detected, under the tested conditions, between the mAb and GST-140-247 (i.e. a C-terminally truncated form of FrC). Therefore, affinity measurements were in agreement with the results previously obtained by ELISA inhibition.

Collectively, our data suggest that, by virtue of its ability to detect overrepresented residues in affinity-selected libraries, PROFILER may provide useful clues for the functional characterization of the epitope. This stems from the ability of next generation sequencing to identify thousands of different inserts, thereby empowering phage display-based methods. This feature is particularly advantageous when using a vector, such as the lambda phage we employed, that can tolerate the expression of a large variety of antigen fragments of different length. Accordingly, in the present study, PROFILER identified a much wider variety of affinity-selected unique sequences, compared with either the traditional immunoscreening-plaque picking method or next generation sequencing of M13 phage libraries. For example, while deep sequencing of the affinity-selected M13 library was able to detect only short (<40 aa long) fragments (Fig. 2C), long fragments were also readily detected by PROFILER, leading to the identification of polypeptides which could better reconstitute the epitope under study. However, it should be noted that, despite their ability to tolerate relatively long fragments, lambda libraries, such as the one used here, might still be unable to completely “cover” some conformational epitopes whose different parts are located in distant areas along the sequence of a large protein. Nonetheless, the PROFILER technology seems capable of readily identifying minimal structures that still retain, at least to some extent, the ability of the whole antigen molecule to react with the test antibody. In fact, when representative “short” and “long” fragments were expressed recombinantly outside of the context of the phage surface, both were capable of binding to the test mAb, although with different affinities. The availability of both “long” and “short” sequences can be useful to expedite further structural analysis based on complex technologies such as x-ray crystallography and nuclear magnetic resonance spectroscopy, particularly when dealing with large conformational epitopes.

In the present study, the availability of an antigen-antibody system whose structural properties are known in detail^16^ allowed us to cross-validate the technique for its ability to correctly identify epitope-containing regions. Notably, PROFILER could provide, in addition, functional insights on antigen-antibody interactions that were not obvious from previous structural analysis^16^. For example, it was possible to identify two short aa stretches (i.e. H248-K254 and S140-G154) that were absolutely or partially required, respectively, for mAb 12C1 binding. Of note, only the first of these aa stretches contained known contact points with the antibody^16^. Nonetheless, further studies involving different antigen-antibody complexes (including “blind systems” with no *a priori* structural knowledge) will be needed to fully assess the usefulness of our approach in the functional characterization of epitopes. In conclusion, the PROFILER technology, by virtue of its ability to identify large numbers of unique antigen fragments, can provide insights into the structural and functional properties of mAb epitopes and may, therefore, be useful in simplifying further structural analysis and in guiding the construction of mutagenized, second-generation libraries.

## Methods

### Monoclonal antibodies and immunological reagents

The murine IgG2b isotype mAb 12C1 was produced and purified by Areta international Srl (Gerenzano, Italy) as described^16^.

### Construction of fHbp var1 gene specific phage displayed libraries

The gene encoding for the fusion antigen GNA2091-fHbp (1314 bp in length), present in the anti-MenB Bexsero^®^ vaccine, was amplified from an expression plasmid used to produce the recombinant antigen (pET-24b+, kindly provided by NVD) using the following primers GNA2091-fHbp_forward 5′-ATGGTCAGCGCAGTAATCGGAA-3′ and GNA2091-fHbp_reverse 5′-TTGCTTGGCGGCAAGGCCGATA-3′.

The amplified product was then purified and randomly digested with DnaseI (Novagen, 69281) and fractionated by 2.5% agarose gel electrophoresis in order to obtain approximately 100–400 bp fragments. These fragments, ligated with specific adapters, were cloned into λKM4 phage vector as described^15^. Recombinant inserts from single clones were analyzed by PCR amplification (primers K47 5′-GGGCACTCGACCGGAATTATCG-3′ and K48 5′-GTATGAGCCGGGTCACTGTTG-3′), as described^15^.

### Affinity selection of phage display libraries and immunoscreening

Affinity selection of the libraries with monoclonal antibody and isolation of positive phage clones were performed as described previously^15^,^17^. Briefly, magnetic beads linked to protein G (Dynabeads^®^ Protein-G, Thermo Fisher Scientific, 10004D) were incubated with library-antibodies mixtures for 1 h at RT under agitation and washed 10 times with 1 ml of washing buffer (1X PBS, 1% Triton, 10 mM MgSO_4_). The bound phages were recovered by infection of LE392 *E. coli* cells (Promega, K9981) added directly to the beads. After a 20-minute incubation, 10 ml of molten NZY-top agar (48 °C–50 °C) was added to the infected cells and poured on NZY plates (Teknova, N1100). The next day, phages were harvested from the incubation plates by gentle agitation with 15 ml of SM buffer (Teknova, S2210) for 4 hours at 4 °C. The phage particles were purified by PEG/NaCl precipitation and stored in SM buffer at 1/10 of the initial volume or used for subsequent selection rounds. Phage pools recovered from the second round of affinity selection on lambda GNA2091-fHbp library with 12C1 mAb were used for Illumina-MiSeq sequencing and for the isolation of individual immunopositive clones by immunoscreening. For immunoscreening assay, LE392 cells were infected with serial dilutions of 12C1 mAb-selected phage pools to obtain plates with 30–300 individual plaques. Plaques from bacterial lawn were blotted onto a dry nitrocellulose filter (Sigma Aldrich, Z6113746) for 2 hr at RT. After blocking with 5% dry nonfat milk in PBS1x for 1 hr at RT, filters were incubated with 12C1 monoclonal antibody (1 μg/ml) for 1 hr at RT with gentle agitation. Filters were then washed with washing buffer (PBS 1x, 0.05% Tween20) and incubated with 1:5000 secondary antibody alkaline phosphatase conjugated anti-mouse IgG (Sigma, A7434) in blocking buffer for 1 hr at RT. Filters were then washed and incubated with 5 ml of substrate buffer containing 330 mg/ml nitro blue tetrazolium, 165 mg/ml 5-bromo-4-chloro-3-indolylphosphate. Reaction was stopped with pouring water. The positive plaques were resuspended in 50 μl of 1x PBS and used for Sanger sequencing as previously described^15^.

### Sample preparation for Sanger sequencing of immunoreactive clones

Plaque picking and sequencing of isolated immunoreactive clones was performed as previously described^12^,^13^,^15^,^17^. Briefly, phage plaques corresponding to immunoscreening reactive clones were picked and resuspended in 50 μl of 1X PBS. Aliquots of phage suspensions (2–5 μl) were added to a PCR mix containing the K47 and K48 primers to amplify the insert-containing region and PCR products were then purified and analyzed by Sanger sequencing using capillary electrophoresis.

### Sample preparation for IlluminaMiSeq sequencing of 12C1 mAb-selected phage pools and Miseq data espression and analysis

Phage pools recovered from the second round of affinity selection on GNA2091-fHbp library were used for Illumina MiSeq sequencing, as previously described^15^.

Briefly phage pools were amplified in *E. coli*, and the lysates were subjected to polyethylene-glycol/NaCl precipitation. Phage suspensions were then added to a PCR mix containing specific the following primers: ≠293: 5′-TCGTCGGCAGCGTCAGATGTGTATAAGAGACAGCGATTAAATAAGG-3′ and ≠294 5′-GTCTCGTGGGCTCGGAGATGTGTATAAGAGACAGGTAATGGGTAAAG-3′.

This PCR added Illumina adaptor subsequences required for an indexing amplification step performed according to the manufacturer’s instructions.

The following primers were used to amplify the inserts of the M13 phage-displayed library and to add Illumina adaptor subsequences: ≠295: 5′-TCGTCGGCAGCGTCAGATGTGTATAAGAGACAGTGCTGCTGGCGG-3′ and ≠296: 5′-GTCTCGTGGGCTCGGAGATGTGTATAAGAGACAGGGCTTGCAGGGAGT-3′.

A LabChip^®^ XT system (PerkinElmer, PN760541) was used for assessing purity, concentration and length of PCR products. Equal volumes of normalized library were then combined and denatured prior to MiSeq sequencing according to manufacturer’s instructions. Sequencing was performed using the Miseq Nano kitv2 and paired end, 150bp-long reads (Illumina, MS-103-1001). MiSeq data expression and analysis was performed as previously described^15^. A fragment was considered to be significantly enriched if its frequency in the selected library exceeded the mean plus 5 standard deviations of the frequency of the fragments in the unselected library. Enrichment factor for each aa residue of the “natural frame” sequences was calculated as the ratio between the occurrence of the residue in the affinity-selected phage population and its occurrence in the unselected library after adding a pseudocount of 1 to the counts for each position.

### Production and purification of fHbp antigenic fragments

Antigenic fragments chosen were subcloned and expressed as recombinant proteins fused to GST: FrC encompassed residues S140-K254; FrN encompassed residues H26-A108; FrS encompassed residues N215-K254.

The DNA sequences corresponding to these fHbp fragments, along with the whole fHbp DNA sequence, were amplified using the following primers:

FrC_forward 5′-ACCGGCTACTAGTTCTTTTGACAA-3′ and FrC_reverse5′-TAATTATGCGGCCGCTCTTGGCGGC-3′; FrN_forward 5′-GGGCGATACTAGTCATAAAGAC-3′ and FrN_reverse 5′-ATAATTAGCGGCCGCTGGCGGTTAA-3′; FrS_forward 5′-TATTATAACTAGTCAACCAAGCCG-3′ and FrS_reverse 5′-AATATATGCGGCCGCCTTGGCGGCAAG-3′; fHbp_forward 5′-TATTATAACTAGTGGAGGGGGTGG-3′ and fHbp_reverse 5′-AATATATGCGGCCGCTTTGCTTGGC-3′.

In addition, the following fragments were also produced: GST-140-247 (i.e. a C-terminally truncated form of FrC) and GST-155-254 (i.e. a N-terminally truncated form of FrC).

For the amplification of the corresponding DNA sequence the following primers were used: GST-140-247_forward 5′-ACCGGCTACTAGTTCTTTTGACAA-3′ and GST-140-247_reverse 5′- TAATTATGCGGCCGCTGCGTATGCCGT-3′; GST-155-254_forward 5′-ATCCTTACTAGTAACCAAGCCGAG-3′ and GST-155-254_reverse 5′-AATATATGCGGCCGCCTTGGCGGCAAG-3′.

Amplified products were purified and subcloned into the bacterial expression vector pGEX(SN), a previously described expression vector^20^ that allows the expression of recombinant proteins as fusions to GST. After induction of the fusion proteins, recombinant fragments were purified from the soluble lysate of bacterial cells by affinity chromatography^12^. Recombinant GST, to be used as a control, was also produced using the same procedures.

### ELISA tests

Immunoreactivity of fHbp recombinant fragments with 12C1 mAb was determined by a previously described ELISA method^21^. Briefly, wells of microtiter plates were sensitized with 5 μg/ml of fHbp recombinant fragments (GST-fHbp, GST-FrC, GST-FrS, GST-FrN, GST-140-247) and GST, as a negative control. 12C1 mAb, 1 μg/ml in antibody buffer (1X TBS, 0,3% BSA), was added to the wells and reacted for 2 hours at 37 °C before the addition of a 1:5,000 dilution of anti-mouse polyvalent IgG conjugated to alkaline phosphatase (Sigma-Aldrich, A7434). Para-nitrophenyl phosphate substrate, (Sigma-Aldrich, N7653) was used to reveal enzymatic activity and absorbance at 405 nm was detected using an automated ELISA reader. Assays were performed in duplicate, and the mean values were calculated.

12C1mAb binding inhibition ELISA assays were performed to assess the ability of the recombinant fHbp fragments to inhibit the reactivity of 12C1 mAb against the homologous antigen fHbp. To this end, 12C1 mAb (1 μg/ml) was pre-absorbed with nanomolar dilutions of GST-fHbp (as a positive control), GST-FrC, GST-FrN, GST-FrS, GST-140-247and GST (as a negative control), and then added to microtiter wells sensitized with the whole protein GST-fHbp (5 μg/ml). Alkaline phosphatase-conjugated goat anti-mouse IgG (Sigma, A7434) was then added at a 1:5000 dilution followed by p-nitrophenyl phosphate substrate (Sigma). Percent of inhibition was calculated by comparing the absorbance value of wells with and without the inhibitors.

### Affinity of antigen-antibody interactions

K_D_ values for complexes formed between mAb 12C1 and fHbp fragments at equilibrium in solution were calculated using an ELISA test, as described^18^,^22^. Briefly, the wells of microtiter plates were coated with 4 different concentrations (3.5, 7, 14 and 28 nM) of each antigen, which consisted of full-length fHbp or of the fragments listed in Table 1. In addition, 7 different concentrations (ranging from 7 to 480 nM) of the antigen were mixed with a fixed concentration of the 12C1 mAb (0.7 nM) and incubated overnight at 4 °C. On the following day, the plates were washed and 100 μl of the 8 antigen-antibody mixtures were dispensed into antigen-coated wells. After incubation at room temperature for 1 h, the wells were washed and alkaline phosphatase-conjugated goat anti-mouse IgG (Sigma) was added, followed by p-nitrophenyl phosphate disodium salt, as described above. Under these conditions, absorbance is proportional to the concentration of free mAb 12C1 added to each well. The results for each antigen were analyzed using the plot described by Friguet *et al.* (ref. 18; Supplementary Fig. 1), which provides a K_D_ value for the complex in solution. Results were expressed as means and standard deviations of the 4 K_D_ values determined for each of the 4 antigen concentrations used to coat the wells.

## Additional Information

**How to cite this article**: Domina, M. *et al.* Functional characterization of a monoclonal antibody epitope using a lambda phage display-deep sequencing platform. *Sci. Rep.* **6**, 31458; doi: 10.1038/srep31458 (2016).

## Supplementary Material

Supplementary Information

## Figures and Tables

**Table 1:** Binding affinities of mAb 12C1 for representative fHbp fragments.

	K_D_ (nM) ± SD	K_D_-fragments/ K_D_-fHbp^#^
GST-fHbp^¥^	0.19 ± 0.03	1
GST-FrC	4.22 ± 0.6	22.78
GST-FrN	79.56 ± 10.11^*^	428.91
GST-FrS	66.09 ± 11.16^*^	356.26
GST-140-247	-^§^	—
GST-155-254	78.41 ± 18.39^*^	422.71

^#^K_D_ variation, compared with the full-length antigen, is shown as x-fold obtained from the ratio K_D_-fragments/K_D_.

-fHbp.

^*^Significantly different (p<0.05) from GST-fHbp by ANOVA and Student-Newman-Keuls test using data from 4 independent experiments.

^§^No interaction was detected under the tested conditions.

^¥^Abbreviations:

GST-fHbp, full-length antigen fused to gluthatione-S-transferase (GST);

GST-FrC, fHbp fragment encompassing residues S140-K254 fused to GST;

GST-FrN, fHbp fragment encompassing residues H26-A108 fused to GST;

GST-FrS, fHbp fragment encompassed residues N215-K254 fused to GST;

GST-140-247, C-terminally truncated form of FrC fused to GST;

GST-155-254, N-terminally truncated form of FrC fused to GST.
