# Supplementary material for: Functional characterization of a monoclonal antibody epitope using a lambda phage display-deep sequencing platform
Source: Sci Rep. 2016 Aug 17;6:31458. doi: 10.1038/srep31458 (PMC4987625; doi:10.1038/srep31458)
Supplement: Supplementary Information [file srep31458-s1.pdf]

Supplementary informations

Functional characterization of a monoclonal antibody epitope using a lambda phage display-deep sequencing platform

Maria Domina[1], Veronica Lanza Cariccio[1], Salvatore Benfatto[2], Mario Venza[3], Isabella Venza[3], Erica Borgogni[4], Flora Castellino[4], Angelina Midiri[2], Roberta Galbo[5], Letizia Romeo[2], Carmelo Biondo[2], Vega Masignani[4], Giuseppe Teti[3,6]*, Franco Felici[7] and Concetta Beninati[1,]

[1] Scylla Biotech Srl, Messina, Italy; [2] Department of Human Pathology, [3] Department of Clinical and Experimental Medicine and [5]Department of Biological, Chemical and Environmental Sciences, University of Messina, Messina, Italy; [4]GSK Vaccines, Siena, Italy; [6]Charybdis Vaccines Srl, Messina, Italy; [7]Department of Biosciences and Territory, University of Molise, Pesche, Isernia, Italy

Supplementary note 1

The probability that a gene fragment is cloned in its natural ("authentic") frame in the amino-terminal region of the D capsid protein is calculated as 1/2x1/3x1/3=1/18, where the first value (1/2) is the probability that the insertion occurs in the forward direction, and the second and third values (1/3) correspond to the probability that the recombinant fragment is in frame at the 5’ and 3’ ends, respectively.

Fig. S1

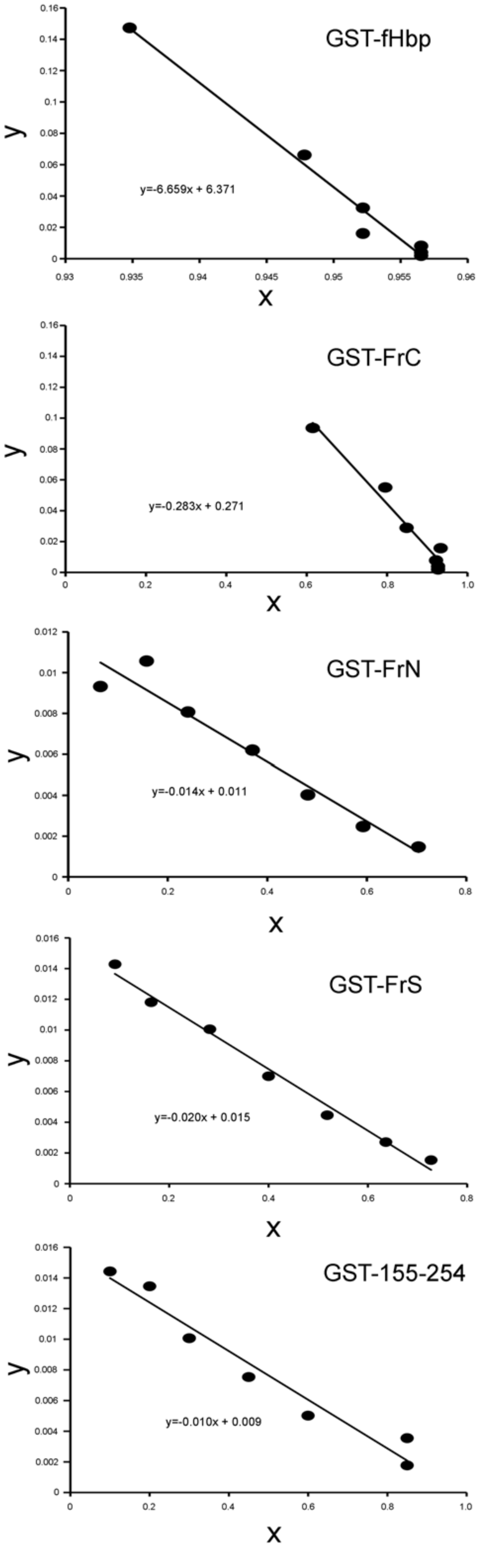
GST-fHbp
y=-6.659x + 6.371
GST-FrC
y=-0.283x + 0.271
GST-FrN
y=-0.014x + 0.011
GST-FrS
y=-0.020x + 0.015
GST-155-254
y=-0.010x + 0.009
x
y

Supplementary Figure 1. Determination of K_D values for mAb 12C1 of the fHbp fragments identified in the present study, as reported in Table 1. The panels show, for each antigen, data plotted on x and y axes, according to the method of Friguet *et al.*[1], as detailed in the Materials and Methods section. x=A_0-A/Ax, where A_0 is the measured ELISA absorbance of the sample containing the mAb alone, and A is the absorbance for a given concentration of free antigen in solution. y is proportional to the fraction of bound antibody in solution, divided by the concentration of free antigen in solution in the mixture:

$$y = \frac{\frac{A_0 - A}{A_0}}{C_{FN} - C_{Ab}\left(\frac{A_0 - A}{A_0}\right)}$$

The experimental data points are fitted to straight line whose slope is equal to -1/K and whose y-axis intercept point is 1/K_D. Shown are representative plots from 4 independent experiments producing similar results.

1 Friguet, B., Chaffotte, A. F., Djavadi-Ohaniance, L. & Goldberg, M. E. Measurements of the true affinity constant in solution of antigen-antibody complexes by enzyme-linked immunosorbent assay. *Journal of immunological methods* **77**, 305-319 (1985).
